# Exploring Family Experiences With Section 504 Plans for Their Autistic Children

**DOI:** 10.1007/s10803-025-06856-2

**Published:** 2025-05-03

**Authors:** RaeAnne Lindsay, Bhabika Joshi, Nehal Abaza, Bridget C. Smith, Saury Ramos-Torres, Rion Humphries, Sarah Demissie, Ana Lucia Hincapie, Isabelle Burakov, Brittney Goscicki, Meghan M. Burke

**Affiliations:** 1 Department of Special Education, Vanderbilt University, Nashville, TN USA; 2 Department of Special Education, University of Texas at Austin, Austin, TX USA

**Keywords:** Sect. 504, Autism, Accommodation, School, Education

## Abstract

Although access to inclusive education should be available for all students with disabilities, the extant literature has focused mainly on access to Individualized Education Programs (IEPs) without addressing access to Sect. 504 of the American Rehabilitation Act. Over 1.38 million students are served by Sect. 504 in public schools, yet little is known about their experiences. Specifically, little is known about the experiences of families of autistic students with Sect. 504; given the heterogeneity of autism, it is likely that many autistic students qualify for Sect. 504 (and not IEPs). The purpose of the study was to explore the experiences of families of children with autism with access to Sect. 504 in school settings. Altogether, 23 families participated in individual interviews about their initial experiences accessing Sect. 504, their input into the Sect. 504 plan, and the implementation of the Sect. 504 plan. Some families reported positive experiences with Sect. 504. When positive experiences occurred, it was largely due to having supportive school professionals. Unfortunately, most participants reported negative experiences with access to Sect. 504 including the school being unwilling to provide accommodations. Implications for future research include the need to develop and test interventions to improve supports under Sect. 504.

Over 1.38 million students are served by Sect. 504 of the Rehabilitation Act of, 1973 in schools across the United States (Office for Civil Rights, 2018). As a civil rights statute, Sect. 504 of the Rehabilitation Act of, 1973 provides accommodations and supports to students with disabilities in any publicly-funded forum, including K-12 schools that receive federal funding. To be eligible for a Sect. 504 plan, a student must have a physical or mental impairment that substantially limits one or more major life activities.

Given its broad eligibility criteria, many students may be eligible for support under Sect. 504 of the Rehabilitation Act of, 1973. For example, among autistic students, autism is developmental disability wherein the traits, strengths, and support needs span a wide spectrum (Masi et al., 2017; Centers for Disease Control and Prevention [CDC], n.d.). While many autistic students may qualify for formal special education services (i.e., an Individualized Education Plan [IEP]), some autistic students may qualify for support (e.g., accommodations) under Sect. 504 of the Rehabilitation Act of, 1973. Like an IEP, Sect. 504 requires students to receive a free, appropriate, public education. Unlike an IEP, students who are eligible for accommodations according to Sect. 504 of the Rehabilitation Act of, 1973 do not need to have written 504 plans, even though written plans are best practice (deBettencourt, 2002). Further, impairments that have an “adverse effect on educational performance” may qualify students for an IEP versus students who have a “mental or physical impairment that affects a major life function” may qualify for accommodations under Sect. 504 of the Rehabilitation Act of, 1973.

Little research has specifically explored the experiences of families of autistic individuals in accessing accommodations accorded by Sect. 504 of the Rehabilitation Act of, 1973. Given the unique profile of families of autistic children in special education (e.g., more likely to advocate and be litigious, Burke & Hodapp, 2016; Zirkel, 2011), it is important to explore experiences with Sect. 504 of the Rehabilitation Act of, 1973 among families of autistic children. By characterizing the experiences of families of autistic children with Sect. 504 of the Rehabilitation Act of, 1973, there can be strategies in policy and practice to improve access to accommodations accorded by Sect. 504 of the Rehabilitation Act of, 1973.

As a first step, it is important to understand family experiences in determining eligibility for Sect. 504 of the Rehabilitation Act of, 1973 for their autistic children. Broadly speaking, to qualify for an autism diagnosis, an individual must meet the two-pronged criteria: persistent deficits in social communication and social interaction across settings and restricted, repetitive patterns of behavior, interests, or activities (American Psychiatric Association, 2022). Professionals, including pediatricians with appropriate training, can provide an autism diagnosis (Hyman et al., 2020). However, the eligibility to qualify for a Sect. 504 plan is much broader than the criteria for autism. Under Sect. 504 of the Rehabilitation Act of, 1973, students may be entitled to an evaluation to identify whether they have a disability (including autism) that substantially limits one or more major life activities. Given the differences in criteria, it is important to explore the eligibility process for autistic children for Sect. 504 plans; by characterizing the eligibility process, there can be a better understanding of how autistic children may qualify for supports.

In addition to exploring experiences with eligibility for Sect. 504 plans, it is also important to identify facilitators in accessing accommodations accorded by Sect. 504 of the Rehabilitation Act of, 1973. Unfortunately, the limited extant research has identified barriers–not facilitators–to accessing accommodations accorded by Sect. 504 of the Rehabilitation Act of, 1973. It is possible that facilitators are the opposite of identified barriers. To this point, prior research has suggested that many education professionals report limited understanding of Sect. 504 of the Rehabilitation Act of, 1973 (Burke & Hodapp 2016; Rodriguez et al., 2021). In particular, school counselors report minimal understanding of Sect. 504 of the Rehabilitation Act of, 1973. Tasked with supporting all students in their school-based academic, career, and social/emotional endeavors, school counselors report struggling to evaluate students for Sect. 504 plans due to their limited time and lack of knowledge about Sect. 504 of the Rehabilitation Act of, 1973 (Goodman-Scott et al., 2020.) In recognition of the limited knowledge counselors have about Sect. 504 of the Rehabilitation Act of, 1973, the American School Counselor Association released a position statement stating that school counselors should not be tasked with managing Sect. 504 plans (American School Counselor Association, n.d.). Research is needed to discern experiences with eligibility and explore whether professional roles and/or knowledge impact access to accommodations provided by Sect. 504 of the Rehabilitation Act of, 1973.

Finally, it is important to identify what characterizes a positive (versus negative) experience with accessing accommodations provided by Sect. 504 of the Rehabilitation Act of, 1973. Although not directly explored in research about students with Sect. 504 plans, in research about family experiences with IEPs, parents of children with disabilities have reported positive and negative experiences (Burke et al., 2022). Positive experiences were often characterized by strong family-professional partnerships. Negative experiences were operationalized as resulting in poor outcomes for children with disabilities, ineffective parent advocacy, and poor family-professional partnerships. It is unclear to what extent, if any, such findings generalize to families of students with Sect. 504 plans. By having a holistic understanding of positive and negative experiences, positive experiences can be replicated and negative experiences can be targeted for intervention.

Given the heterogeneity in autism, it is likely that many autistic students may qualify for Sect. 504 plans. Yet, little is known about the experiences of families of autistic children with Sect. 504 plans in school settings. By exploring their experiences, there can be a more comprehensive understanding of ways to improve access to accommodations accorded by Sect. 504 of the Rehabilitation Act of, 1973 among autistic students. For this study, the research questions were: Among families of school-aged autistic children, (1) What are their experiences in becoming eligible for Sect. 504 of the Rehabilitation Act of, 1973?; (2) What are the facilitators to accessing supports under Sect. 504 of the Rehabilitation Act of, 1973?; and (3) What differentiates positive and negative experiences with Sect. 504 of the Rehabilitation Act of, 1973?

## Method

### Research Design

Given the limited research about family experiences with Sect. 504 of the Rehabilitation Act of, 1973 (Zirkel, 2023), it was appropriate to conduct an exploratory study of family experiences. Specifically, individual qualitative interviews were conducted with families of autistic children who had Sect. 504 plans to explore their experiences with Sect. 504 of the Rehabilitation Act of, 1973 in school settings. The first author spearheaded the study. The second author assisted with data analysis and writing. The remaining authors assisted with data collection and analysis. The last author conceptualized the study and helped write the manuscript.

### Researcher Identity and Positionality

Our research team was comprised of ten individuals including one professor, one assistant professor, two doctoral students, three master’s students, and three researchers with disabilities (including one with autism). In addition, three team members had family members with disabilities. Everyone on our team had experience working with individuals with disabilities, including autism. Collectively, our team believes that every student with a disability—including autistic students—are entitled to meaningful accommodations and supports in school settings. Throughout the project, our team met to discuss our values and biases.

### Participants

This study is part of a larger project to explore the experiences of students with disabilities and their families with Sect. 504 plans in K-12 schools. For this study, the inclusionary criteria required each participant to be: the caregiver of an autistic child who applied for and/or received school supports under Sect. 504 of the Rehabilitation Act of, 1973. Altogether, there were 23 participants. Participants could complete the research procedures in English or Spanish; one participant completed the study in Spanish and the other 22 participants completed the study in English. On average, participants were 42.4 years old (*SD* = 6.60). The majority of the participants was white (*n* = 18, 85.7%). Most participants were mothers (*n* = 20). Participants reflected 13 states. The children of the participants ranged from 3 to 17 years of age. Of the 23 participants, 21 participants’ children attended public schools. See Table 1.

**Table 1:** Participant demographics

Name	Role	Age	Marital Status	Education	Income	Race/Ethnicity	Child age	State	Type of school
Cindy	Mother	41	Married	High school	$30–49,999	Latino	17	IL	Public
Mary	Mother	44	Single	Graduate degree	$70–99,999	White	13	IL	Public
Georgia	Mother	40	Married	Graduate degree	>$100,000	White	7	VA	Public
Kimberly	Mother	40	Married	College degree	>$100,000	White	15	MD	Public
April	Mother	36	Married	College degree	>$100,000	White	3	NC	Public
Makayla	Mother	40	Married	Graduate degree	>$100,000	White	12	CA	Public
Samantha	Mother	43	Married	Graduate degree	>$100,000	White	8	GA	Public
Amelia	Grandma	53	Married	College degree	>$100,000	White	8	MO	Public
Abigail	Mother	38	Single	Graduate degree	>$100,000	White	10	CA	Public
Deirdre	Mother	61	Single	Some college	$70–99,999	White	16	WA	Public
Amy	Mother	46	Married	Some college	>$100,000	White	15	OH	*Homeschool
Emily	Mother	39	Married	Some college	$70–99,999	White	12	WA	Public
Molly	Mother	47	Married	Some college	$50–69,999	White	12	CA	Public
Charlotte	Mother	43	Married	Graduate degree	$50–69,999	White	9	MO	Public
Emma	Mother	44	Single	College degree	$30–49,999	Native American	11	CA	Public
Sophia	Mother	36	Married	College degree	$70–99,999	White	8	OR	Public
Karen	Mother	40	Married	Graduate degree	>$100,000	White	9	MO	Public
Taylor	Mother	36	Married	College degree	>$100,000	White	7	OH	Public
Mayci	Mother	41	Married	College degree	>$100,000	White	11	IL	Public
Violet	Mother	41	Married	Graduate degree	$30–49,999	White	14	IN	Public
Lisa	Mother	39	Married	Some college	$50–69,999	White	12	OH	Public
Stella	Mother	42	Married	Graduate degree	>$100,000	White	12	CT	Private
Melinda	Mother	42	Single	College degree	>$100,000	Asian	15	OR	Public

*Previously attended a public school

### Recruitment

Participants were recruited in a variety of ways. Namely, there was a recruitment flyer in English and Spanish. The flyer was disseminated to disability organizations, family organizations, and parent support groups throughout the United States. To aid in recruitment, each participant received a $60 gift card. Specifically, each participant received a $50 gift card after completing the interview and a $10 gift card after completing the member check. Recruitment ended after we determined a redundancy of themes.

### Procedures

All research procedures were approved by the University Institutional Review Board. Upon reviewing the recruitment flyer, interested individuals read the consent form and provided consent via RedCap, an online survey platform. After providing consent, individuals completed a brief survey. Following the survey, a research team member contacted the individual to arrange the interview at a participant-preferred date and time. The participant chose whether to conduct the interview via phone or over Zoom; all participants chose to conduct the interview over Zoom. The research coordinator trained all interviewers to conduct interviews with fidelity.

At the beginning of the qualitative interview, the interviewer explained their connection to disability to establish rapport with the participant (O’Toole, 2022). Then, the interviewer conducted the interview. Interviews lasted, on average, 46 min (ranging from 21 min to two hours and 26 min). All interviews were recorded and transcribed verbatim. After completing the interview, an artificial intelligence mechanism created a summary of the interview. The interviewer reviewed the summary to ensure its accuracy and completeness. The summary was e-mailed to the participant to check its: accuracy, completeness, and whether anything needed to be added or changed. For this study, 16 participants responded to the member check. Minor changes were asked from the participants who responded. Such changes included correcting the gender identity of the participant and correcting the names of school staff.

### Fidelity to the Interview Protocol

An independent researcher reviewed each qualitative interview to determine its fidelity to the interview protocol (i.e., whether each interview question was asked of each participant). Fidelity to the interview protocol was 100%.

### Translation

The study materials (e.g., recruitment flyer, consent form) were translated by independent researchers into Spanish (Brislin, 1970). The research team included two researchers who identified as Latina and native Spanish speakers. Notably, the interview protocol was piloted in Spanish twice before commencing the study. For this study, one interview was conducted in Spanish. A bilingual research team member conducted the interview. The interview was transcribed verbatim in Spanish. Data analysis was conducted in Spanish by the two researchers who identified as Latina.

### Measures

#### Survey

After providing consent, the participant completed a 5–10-minute survey. In the survey, the participant answered demographic questions (e.g., gender, race, ethnicity, socioeconomic status) and close-ended questions about their experiences with the Sect. 504 of the Rehabilitation Act of, 1973 (e.g., whether their child had a Sect. 504 plan, whether the family had filed a procedural safeguard about Sect. 504 of the Rehabilitation Act of, 1973). The survey responses were used to characterize the sample and identify patterns in the data.

#### Interview Protocol

The qualitative interview protocol was developed based on legislation and research about Sect. 504 of the Rehabilitation Act of, 1973 (e.g., Zirkel et al., 2023; Burke & Hodapp 2016). Individuals with disabilities and families reviewed the interview protocol and provided feedback. The protocol was piloted with two families of children with disabilities; minor changes were made. For example, based on the piloting, we showed the participants the interview questions on the zoom screen so they could hear and read the questions throughout the interview. The protocol was comprised of three sections: (1) initial experience in accessing supports under Sect. 504 of the Rehabilitation Act of, 1973, (2) the development of the Sect. 504 plan and (3) the implementation of the Sect. 504 plan.

### Data Analysis

To analyze the data, we used emergent design (Patton, 2002). First, we familiarized ourselves with the data by independently reading each transcript multiple times (Tesch, 1990). Then, we openly coded in response to our research questions. Open coding initially occurred with six transcripts as research suggests that most themes can be identified within six interviews (Guest et al., 2006). We met to discuss and compare our codes. A codebook was then created with our collective codes. We returned to the same six interviews using the codebook to identify whether there were any new codes; we revised the codebook accordingly. We continued to use the updated codebook to code the remaining interviews. We met to compare our codes for each interview. If we had different codes, we reconciled the codes until we were in agreement.

For example, with respect to eligibility for Sect. 504 of the Rehabilitation Act of, 1973, there were several codes. Such codes included: private evaluation, school evaluation, medical diagnosis, no evaluation, formal data, informal data, formal assessment, and informal assessment. Codes were grouped into categories (e.g., formal evaluations, informal evaluations, no evaluation). Categories were clustered into themes: private evaluation showing a medical diagnosis; school refuses to conduct an evaluation; and school conducts an evaluation.

### Trustworthiness

Several methods were used to ensure the trustworthiness of the data collection and analysis procedures (Brantlinger et al., 2005). For example, peer debriefing was used throughout data collection and analysis. Our team met weekly to discuss the project, identify themes, and discern potential patterns in the data. Also, data triangulation was used between the surveys and interview data. Two-level member checking was also used in this project. As a first-level member check, at the end of the interview, the interviewer summarized what was said during the interview. As a second-level member check, each participant was sent a summary of their interview to check for accuracy.

### Findings

#### Family Experiences in Accessing Sect. 504 of the Rehabilitation Act Of, 1973

Participants reported diverse experiences in qualifying for accommodations accorded by Sect. 504 of the Rehabilitation Act of, 1973. Experiences included: receiving private evaluations showcasing a medical diagnosis; the school conducting an evaluation; and the school not conducting an evaluation. See Table 2.

**Table 2:** Themes

Research Question	Themes
Family Experiences in Accessing Sect. 504 of the Rehabilitation Act of, 1973	
	Private Evaluations Showcasing a Medical Diagnosis
	School Conducted an Evaluation for Eligibility for Sect. 504 of the Rehabilitation Act of, 1973
	School Professionals Refused to Conduct an Evaluation
Facilitators to Access Accommodations Per Sect. 504 of the Rehabilitation Act of, 1973	
	Responsive school staff
	Parent knowledge and advocacy skills
	Social capital including family and friends
Differences Between Positive and Negative Experiences	
	Positive experiences: Supportive networks
	Negative experiences: Unsupportive school professionals
	Negative experiences: Taking advantage of lack of parental knowledge about Sect. 504 of the Rehabilitation Act of, 1973
	Negative experiences: Negative effects on autistic children
	Negative experiences: Inexperienced school staff
	Negative experiences: Gender discrimination

#### Private Evaluations Showcasing a Medical Diagnosis

Several participants reported sharing their private medical evaluations showcasing the autism diagnosis with school professionals. Private evaluations were conducted by neuropsychologists, psychologists, pediatricians, and therapists. For example, Violet reported that she sought a private neuropsychological evaluation for her daughter. The evaluation led to an autism diagnosis for her daughter (now aged 14). Violet stated, “The reason [the school] finally did testing is because we did full battery [testing] through an outside psychologist that found all these things, you know, found that she was autistic.” Some participants reported a need to secure private evaluations because the school refused to initiate an evaluation. Mary, the mother of an 11-year-old boy, explained her experience: “I gave [his educator] his [neuropsychological] evaluation, which gave the diagnosis of autism and mood dysregulation disorder. And [the school] didn’t say, ‘Hey, you have a child that’s disabled, and we want to make sure that we’re supporting him in the best ways.’…they [the school professionals] should have said we can assess him and help support. And they never did that. So, I’m frustrated for that. But it was me that led that–not the school at all.” Other participants reported that the school accepted the outside evaluation and did not pursue their own testing. While Lisa shared her son’s medical diagnoses with the school, the school refused to evaluate whether the child needed a Sect. 504 plan: “I presented [the school professionals] with letters from the doctors and psychs [psychologists], and everyone that had given us the diagnoses. They wrote letters stating like I think [my daughter] should have a 504…once we presented the school with those letters, that’s when they decided they weren’t even going to do their own assessments. They were just going to go off of that.”

#### School Conducted an Evaluation for Eligibility for a Sect. 504 Plan

Some participants reported receiving holistic evaluations including assessments from varied professionals (e.g., the school psychologist, speech therapist, occupational therapist). Other participants reported inadequate evaluations reflecting an assessment with only one school professional. Regarding the former, Emily reported that the school professional conducted a comprehensive evaluation. Specifically, Emily reported that the teacher told her: “Okay, we’re gonna do a full evaluation. [The school professional’s] gonna look over things. They’re gonna watch your kid for a little bit.” Unlike Emily, Kimberly reported having an inadequate evaluation. She stated that although the school assessed her child academically, her child needed to be assessed socially: “[My child] was evaluated in academics and that was pretty much it even though I said it was more than that. Again, I kind of had the struggle with getting anybody to take seriously, because my child is a high masker at that time. We didn’t know that they were also autistic”.

#### School Professionals Refused To Conduct an Evaluation

Some participants reported that the school professionals refused to evaluate their child for a Sect. 504 plan. Molly submitted an evaluation request to the principal, resource teacher, special education director, and the general education teacher. Even though she received a formal letter stating the approval to initiate the process, the evaluation never happened. Molly recollected, “[The school professionals] called me in for a meeting to discuss [the evaluation]. And all those people that I mentioned, plus a couple more, were in the initial meeting and where they denied starting access. They didn’t jumpstart the process. They didn’t assess the student for anything…. They denied any kind of testing.”

### Facilitators To Access Accommodations Per Sect. 504 of the Rehabilitation Act Of, 1973

Participants reported several facilitators in accessing supports under Sect. 504 of the Rehabilitation Act of, 1973. Facilitators included: responsive school staff; parent knowledge and advocacy skills; and social capital (including family and friends).

#### Responsive School Staff

Several participants discussed the importance of responsive school staff in ensuring access to supports under the auspice of Sect. 504 of the Rehabilitation Act of, 1973. Participants defined school staff as general education teachers, principals, assistant principals, occupational therapists, psychologists, counselors, and/or superintendents. Emily shared that her experience hinged on the responsiveness of the general education teacher: “The teacher knew step A, B, and C when first trying to access a Sect. 504 plan. She would teach us each step and shared that there was a paper that we needed to fill out to begin the process. We filled it out than gave it back to her so that she could pass it along. I didn’t have to deal with having to talk to each individual person at the school to figure out how to start. She did all the behind the scene work.” Deirdre reported that the superintendent supported her child’s need for a Sect. 504 plan: “I shared the data that we had from the medical providers with the superintendent. I shared that I could understand the hesitation on an Individual Education Program [IEP] but that there is nothing that says that we couldn’t have a Sect. 504 plan. The superintendent and the rest of the school were very kind and worked well together, which resulted in us receiving a Sect. 504 plan.”

#### Parent Knowledge and Advocacy Skills

Many participants commented that their knowledge about Sect. 504 of the Rehabilitation Act of, 1973 and their advocacy skills facilitated their access to Sect. 504 plans. Participants characterized knowledge and advocacy skills in several ways including: researching Sect. 504 of the Rehabilitation Act of, 1973, leveraging external evaluations and therapy reports, and having prior experience with requesting accommodations. Sophia shared an online resource, A Day in Our Shoes, that she found beneficial when discussing accommodations with the school. A Day in Our Shoes is a website that explains to caregivers their rights accorded by Sect. 504 of the Rehabilitation Act of, 1973; the website offers articles, toolkits, and videos. The website also offers advocacy trainings and transition workshops. Sophia reported, “They [the school] had a list of the typical accommodations that they did. It was more generic than anything, but I also had a list from, A Day in Our Shoes, of more specific accommodations to ask for. The discussion then changed to them [school] sharing what they couldn’t do from the list to what they could do to make sure the [child’s] needs were met.” Stella discussed how her and husband conducted legal research before their meeting with the school. Stella recalled her experience,

We did some legal research ahead of time. We have some experience accommodations—since my husband is a teacher and I am a social worker—but we were not sure how to begin. We wrote a statement—like a one line typed out statement—requesting our child to be evaluated, and [we also wrote] what we would do if they refused [our request]. We wanted this statement because we had a feeling that the school was just going to flat out say no to our request, which they did. We gave them the statement saying that we disagreed with their denial and we would be pursuing an IEP at their cost. The meeting ended then and we were later asked to come back in to discuss this further. What had happened was the school team that we had met with, took our statement to the board and the board overruled them saying that the school would evaluate our child and while that was going on, they offered us a Sect. 504 plan.

April reported that she had a Sect. 504 plan herself. As a result, she knew how to advocate for her child: “I am an advocate…It wasn’t something that I had not been through before. I knew what I could ask for and what our rights were. I definitely think that was an advantage.” Melinda reported that she belonged to a network of advocates called Activate Your Advocacy (AYA). Melinda said, “I was invited to be a part of Activate Your Advocacy…I feel very empowered after being a part of their program. Growing up the way I did, fighting for myself, then becoming a nurse gave me a unique experience. I’m a fighter and that crossed over into fighting for my child getting the supports that they needed.”

#### Social Capital Including Family and Friends

Several participants reported that social capital (characterized as family and/or friends) helped them access Sect. 504 plans. Mayci reported having friends who also had children with disabilities. With her friends, she discussed how to access supports under Sect. 504 of the Rehabilitation Act of, 1973: “Friends made it easier to receive a Sect. 504 plan. There are other families that have the same diagnosis, and I am in close contact with them. So, we kind of get a heads up about what may be coming down the road for my child.” Charlotte discussed how the school did not make it easy to access services but having friends that are occupational therapists or being connected to other families in the school system aided them in receiving services. Charlotte reported, “We had friends who are [occupational therapists] or we got put in touch with other people within the school system that had experience with doing these evaluations. They were able to guide us and say, ‘Well, you need to explain this or that to make your case.’”.

### Differences Between Positive and Negative Experiences

Most participants characterized their experiences in accessing accommodations per Sect. 504 of the Rehabilitation Act of, 1973 as negative. Of the few participants who characterized their experiences as positive, the participants attributed their experiences to having supportive networks. Negative experiences were characterized by: unsupportive school professionals, taking advantage of lack of parental knowledge about Sect. 504 of the Rehabilitation Act of, 1973, negative effects on autistic children, inexperienced school staff, and gender discrimination.

#### Positive Experiences: Supportive Networks

The few participants with positive experiences attributed their experiences to having supportive networks with school professionals and/or other families of children with disabilities. Regarding the former, Charlotte reported that, initially, the school professionals were resistant to providing her son with a Sect. 504 plan. However, the school professionals changed their minds after hearing from Charlotte. This receptiveness facilitated a positive experience for the family:

The school] was pretty resistant at first to determine eligibility, but then, once we got past that, they were there. It really felt like they switched gears. It was a lot more cooperative and collaborative at that point. They really stopped to think about how they could help and support my child since we had a plan. They thought through what was reasonable and what would actually help.

Georgia shared that her daughter’s teacher was instrumental in initiating her daughter’s evaluation. Georgia reflected that she was resistant to the evaluation but her teacher supported her during the process:

My daughter was having a lot of like behavior problems and things like that. At first, I was like, ‘Oh, it’s just kindergarten’ and ‘She’s only five’. But it was her teacher who said later in the year that she was wondering if my daughter had ADHD [Attention-deficit/hyperactivity disorder] because of the signs she was seeing. I was kind of resistant at first but the teacher really talked to me about how we should work with the school to see if there could be any supports that would benefit my daughter. The teacher and school really supported us to find supports.

With respect to support from other families of children with disabilities, Kimberly reported that knowing other families of children with Sect. 504 plans made her experience more positive:

What really helped us was talking to other people. Whether it was people that I knew from the same school system or just others that we connected with in the community. Because trying to understand federal law alone is difficult. We learned what kind of questions to ask or [other families of children with disabilities] gave us templates to follow. It was incredibly helpful and it’s something that I wish there was more of.

Mayci reported about the importance of having friends who also have children with disabilities. Mayci stated, “Definitely friends who have the same diagnosis that I am in close contact with. It gives us a heads up on what the future may look like. Having a community is key.”

#### Negative Experiences: Unsupportive School Professionals

Several participants reported that their experiences with Sect. 504 of the Rehabilitation Act of, 1973 were negative because of unsupportive school professionals. Mary shared that even with medical records documenting her child’s ADHD and autism diagnoses, the school was “not supportive of an IEP plan.” When she pursued a Sect. 504 plan for her child, the school was still “resistant a lot of times” on providing accommodations. Dismayed, she shared that the school “really looked at him [son] with just a behavior child and not that he had a true disability.” Other participants similarly reported unsupportive school professionals leading to feelings of disrespect, burnout, and frustration. Sophia reported frustration that the school professionals “kept on saying…that [Sophia] wasn’t an adequate source for the knowledge about my own child.” She shared that the school “talked down to [her]” and “devalued [her] information by saying that [she] wasn’t adequately [trained].”

#### Negative Experiences: Taking Advantage of Lack of Parental Knowledge About Sect. 504 of the Rehabilitation Act Of, 1973

Some participants reported feeling that school professionals took advantage of them because of their lack of knowledge about Sect. 504 of the Rehabilitation Act of, 1973. Specifically, some participants reported that school professionals talked dismissively to them, intimidated them, and/or ignored their requests. Emma reported that, initially, she did not understand Sect. 504 of the Rehabilitation Act of, 1973; recognizing her lack of knowledge, she reported that the school did not appropriately support her son. Karen and Stella both reported feeling “gaslit” by school professionals. Specifically, Karen reported that when she requested educational accommodations for her daughter, the school professionals: “thought we were crazy for asking for help for our daughter.” When Stella tried to advocate for her child, the school board pushed back and denied access to mediation and due process, even though she was legally entitled to those procedural safeguards. She described the process as “hostile, very contentious, very intimidating.” Additionally, she added that the board members made her feel “dehumanizing…[the school board] make you think you’re crazy. They make you second guess your child.”

Some participants reported that the school took advantage of them by refusing to communicate with families. Such actions included: school professionals refusing to put communication in writing; no communication about student progress; no dialogue about initiating evaluations for Sect. 504 plans; no information about data being collected for the student; and a refusal to share data. Taylor explained that when she reached out to inquire about her child’s progress at school, “[her] questions weren’t answered so [she felt] uncertain about how the supports actually benefitted [her son].” Similarly, Molly reported that when she reached out after her son was denied for services, “[her] emails were ignored.” Karen reported that Sect. 504 meetings occurred between “the counselor, the final coordinator, and [her] daughter” without any invitation for her (the mother) to attend. Similarly, Melinda reported that the school did not invite her to attend Sect. 504 meetings: “school insisted on having a meeting without [their] presence.”

#### Negative Experiences: Negative Effects on Autistic Children

Within negative experiences, several participants reported negative effects on their autistic children. Negative effects included: inefficient transitions to higher grade levels and ineffective implementation of the Sect. 504 plan. Cindy reported that her daughter did not receive appropriate educational supports when she transitioned to high school stating she “went a year without accommodations”. Accordingly, Cindy reported that her daughter experienced high levels of “stress” and “no access to therapy.” Several participants reported negative consequences of the school’s non-adherence to Sect. 504 plans. Following lack of initiative from the school and denied access to supports accorded by Sect. 504 of the Rehabilitation Act of, 1973, Karen shared that their daughter was “diagnosed with anxiety…and started [taking] SSRI medication.” While astonished that their daughter was on anxiety medication at seven-years-old, Karen understood that their daughter needed the support system that the school was not providing for her. Even after she started the process of accessing a 504 plan, the procedures took so long that her daughter was “showing signs of dysregulation.”

### Negative Experiences: Inexperienced School Staff

Some participants reported negative experiences due to school professionals lacking experience and knowledge about autistic students and/or Sect. 504 of the Rehabilitation Act of, 1973. With respect to general education teachers, Mary reported that they “don’t know how to support some of our neurodivergent students.” Other participants reported that school professionals did not understand Sect. 504 of the Rehabilitation Act of, 1973. Makayla reported that school professionals misunderstood the purpose of a Sect. 504 plan. Makayla reported that, after being denied eligibility for a continuing IEP, she requested a Sect. 504 plan. School professionals also rejected the Sect. 504 plan, claiming that “they couldn’t give a 504 until [my son] was exited completely from special ed through the IEP.” Beyond eligibility, some participants reported that school professionals did not understand how to provide individualized accommodations. Emily reported that the school professionals insisted on providing a premade list of accommodations even after she advocated for individualized accommodations.

#### Negative Experiences: Gender Discrimination

Some participants reported experiencing discrimination due to their gender. Melinda reported gender discrimination during her son’s evaluation. She acknowledged that as a woman, her gender made it more difficult to access services, noting that she did not “think people always take women seriously.” While she mentioned that her identity as a White woman has given her privilege, her biracial child faced challenges in accessing accommodations under Sect. 504 of the Rehabilitation Act of, 1973. Other female participants also reported that school professionals were less likely to respond to their advocacy. Simply put, Karen reported she was taken “less seriously” than fathers.

## Discussion

This study is an important jumping off point to explore the experiences of caregivers of students with autism with Sect. 504 of the Rehabilitation Act of, 1973. We had four main findings. First, family-school partnerships matter. Specifically, if there were supportive partnerships, families reported positive experiences with accessing supports accorded by Sect. 504 of the Rehabilitation Act of, 1973; conversely, if the partnerships were negative, participants characterized their access to supports accorded by Sect. 504 of the Rehabilitation Act of, 1973 as negative. Over the past several decades, extant literature and policy have consistently reinforced the importance of high-quality, positive family-professional partnerships among autistic students with IEPs (Goldrich Eskow et al., 2018; Sreckovic et al., 2021). Further, the research has suggested that, compared to families of children with other types of disabilities, families of autistic children report worse partnerships with school professionals (Burke & Hodapp, 2016; Decker, 2012; Zirkel, 2011). Many mothers of autistic youth report “fighting” for services and perceiving schools to underestimate support needs (Fowler & O’Connor, 2021). When families and school professionals do not partner with one another, there can be negative implications for autistic students. For example, parents and teachers often report varying degrees of autistic traits of their students resulting in some autistic students not receiving needed services in schools (Putnam et al., 2024). This study contributes to the literature by generalizing the importance of partnerships to families of autistic children with Sect. 504 plans.

Building on this finding, this study tells us that practitioners not only need to be competent about Sect. 504 of the Rehabilitation Act of, 1973 but also need to demonstrate warmth towards students with disabilities. The importance of competence and warmth among professionals is not new. Seminal research has documented that professionals must not only be competent (i.e., knowledgeable about their subject area) but also warm (i.e., kind to those they serve) (Fiske et al., 2007). This finding suggests that school professionals need training about Sect. 504 of the Rehabilitation Act of, 1973 in combination with instruction about how to meaningfully and respectfully partner with families and their autistic children.

Second, social capital—including friends and family—facilitated positive experiences with Sect. 504 of the Rehabilitation Act of, 1973. Broader literature on social capital theory (Bourdieu, 1986) suggests that access to family, friends, and/or community can improve one’s quality of life. Among families of children with disabilities, peer support has frequently been heralded as critical (Dodds, 2021). With respect to families of autistic children, friendships with other families of children with autism has been noted as an important support (Goedeke et al., 2019; Lee et al., 2024; Roffeei et al., 2015). Indeed, families of autistic children report preferring to hear from other families of individuals with autism specifically (not families of children with other types of disabilities) given their shared experiences (Burke & Hodapp 2016).

Relatedly, social capital was one way in which participants developed knowledge and/or advocacy skills in the context of Sect. 504 of the Rehabilitation Act of, 1973. While prior studies have explored the limited knowledge about Sect. 504 of the Rehabilitation Act of, 1973 among teachers (O’Connor et al., 2016), principals (Roberts & Guerra, 2017), and families (Besnoy et al., 2015; Connor & Cavendish, 2018), little is known about how individuals—including families—develop knowledge about Sect. 504 of the Rehabilitation Act of, 1973. This finding suggests that peer support may facilitate knowledge and advocacy skills. When considering developing interventions to improve knowledge and advocacy among families, this finding may suggest a cohort-model enabling families to meet other families as a way to develop their competence.

Third, this study highlighted that, when not provided with a comprehensive evaluation and/or paired with unexperienced or unwilling professionals, autistic children may experience poor outcomes. With respect to families of autistic children with IEPs, the research suggests that, when children do not receive appropriate services, they experience worse outcomes (Reiman et al., 2010; Sanderson, 2023). This study suggests that poor outcomes extend to autistic children under the auspice of Sect. 504 of the Rehabilitation Act of, 1973. Given that there are many implementation problems with Sect. 504 of the Rehabilitation Act of, 1973 (Lewis & Muñiz, 2023), this finding is particularly concerning. Future research is needed to understand whether eligibility and implementation problems longitudinally impact children including (but not limited to) their academic, social-emotional, and functional outcomes.

Finally, this study suggests that evaluation experiences varied for Sect. 504 of the Rehabilitation Act of, 1973. For the participants who were denied an evaluation, there could be concerns about the delay in accessing needed supports. Previous research has underscored the consequences of misidentification and missed identification (Mitchell, 2017; Raj, 2016) among autistic children. Such consequences include emotional turmoil and poor child outcomes. In comparison, timely diagnoses of autistic children lead to faster access to intervention and services (Penney et al., 2022). More research is needed to ensure that autistic children are being efficiently identified and supported by Sect. 504 of the Rehabilitation Act of, 1973. Future research about evaluations and eligibility for accommodations accorded by Sect. 504 of the Rehabilitation Act of, 1973 should reflect the vague laws surrounding eligibility. Indeed, eligibility for accommodations accorded by Sect. 504 of the Rehabilitation Act of, 1973 is made on a case-by-case basis; thus, the process will not look the same for every student (for example, assessments may or may not be conducted as part of the eligibility process). Also, Sect. 504 of the Rehabilitation Act of, 1973 does not obligate school professionals to use (or not use) external evaluations. Further, with respect to disagreements, Sect. 504 of the Rehabilitation Act of, 1973 does not have dispute-resolution processes, only procedural safeguards. Thus, when considering options to resolve disputes, it is important to consider what is and is not afforded by Sect. 504 of the Rehabilitation Act of, 1973.

The delay in receiving an evaluation and/or services may be due to masking. As shown in this study, some participants reported their autistic children masked their disability. Autistic students who have less support needs (Shattuck et al. 2009) and engage in camouflaging behaviors to present non-autistic personas are more likely to have missed or later diagnoses (Lai et al., 2015), along with greater mental health challenges (Bernardin et al., 2021). Future research is needed to disentangle the effect of masking on Sect. 504 of the Rehabilitation Act of, 1973 implementation and student outcomes.

### Limitations

While this study contributes to the sparse literature about families of autistic children with Sect. 504 plans, there are a few limitations. First, the participants were primarily white and highly educated. Thus, there is limited transferability of the findings to the broader population. Future research should be conducted with families of color to better understand their experiences with Sect. 504 of the Rehabilitation Act of, 1973. Second, the data were cross-sectional. It is possible that there are changes over time with experiences with Sect. 504 of the Rehabilitation Act of, 1973; longitudinal research is needed to understand how familial experiences with Sect. 504 of the Rehabilitation Act of, 1973 ebb and flow over the child’s educational lifespan. By determining whether child age impacts experiences with Sect. 504 of the Rehabilitation Act of, 1973, targeted interventions can be developed in a developmentally appropriate way.Third, the study was limited to family interviews and surveys. Future research may include an evaluation of the Sect. 504 plan itself (if it was written) and/or other perspectives to develop a more holistic understanding of experiences with Sect. 504 of the Rehabilitation Act of, 1973.

### Directions for Future Research

Future research should consider including the perspectives of autistic students. In alignment with the ‘Nothing about us, without us’ mantra of the disability rights movement (Charlton, 1998), it is critical to consider the lived experiences of autistic students. Further, self-determination is a key predictor of positive school and post-school outcomes (Shogren et al., 2015). Future research should include collecting data from autistic students to understand not only their experiences with Sect. 504 of the Rehabilitation Act of, 1973 but also their self-advocacy in the process of accessing supports.

In addition, future research should consider biosocial methods to examine the effect of the Sect. 504 of the Rehabilitation Act of, 1973 process on families. Participants often discussed emotional experiences including frustration, stress, and anxiety in response to negative interactions with school professionals. In research about mothers of children with developmental disabilities, including autism, Burke and Hodapp (2014) found that maternal stress significantly increased when there were poor family-school partnerships. Biosocial methods could help discern the relation between biological and social factors during the Sect. 504 of the Rehabilitation Act of, 1973 process. For example, researchers could collect cortisol and heart rate samples among families of autistic children before, during, and after Sect. 504 meetings with school professionals. They could triangulate the biological data with survey data from families about the quality of their partnership with educators. Such research could inform whether the Sect. 504 of the Rehabilitation Act of, 1973 process impacts parent well-being.

### Implications for Policy and Practice

Practitioners should be offered ways to learn more about Sect. 504 of the Rehabilitation Act of, 1973, autism, and accommodations. To this end, school districts may consider offering different ways to educate practitioners. Such methods may include professional development, access to practitioner journals, and/or policy briefs explaining the law and its implementation in schools. By educating practitioners about Sect. 504 of the Rehabilitation Act of, 1973 and the responsibilities of the school system, there may be an improved implementation process for autistic students with Sect. 504 plans.

Implications also exist for families. Parent Training and Information Centers (PTIs) are federally-funded centers that educate and empower families of children with disabilities. There is at least one PTI in every state. While PTIs mostly offer training about IEPs, this study suggests that training is also needed about Sect. 504 plans. To this end, PTIs may consider increasing their trainings and workshops to include rights accorded to families by Sect. 504 of the Rehabilitation Act of, 1973.
